# Ultrasound-Guided Collagenase Injection Therapy of Recurrent Plantar Fibromatosis: A Case Report

**DOI:** 10.1177/24730114231201161

**Published:** 2023-10-20

**Authors:** Antje L. Greenfield, Adan Bello Baez, Casey Jo Humbyrd

**Affiliations:** 1 Department of Radiology, Section of Musculoskeletal Radiology, Penn Medicine University City, Philadelphia, PA, USA; 2 MSK Radiology, Hospital Universitario Nuestra Señora De La Candelaria, Tenerife, España; 3 Department of Orthopedic Surgery, Division of Foot and Ankle Surgery, Penn Musculoskeletal Center–Penn Medicine University City, Philadelphia, PA, USA

**Keywords:** plantar fibromatosis, collagenase Clostridium histolyticum, ultrasonographic guidance

## Introduction

Plantar fibromatosis, also known as Ledderhose disease, is benign nodular hyperplasia of fibroblastic connective tissue of the plantar fascia.^7^

Patients experience slowly enlarging palpable nodules located in the soft tissues of the plantar foot. These masses can be asymptomatic, especially when they are small. In some patients, the fibromatous nodules become quite large and painful, resulting in difficulty walking or fitting shoes.^4^

These lesions are histologically similar to Dupuytren’s contracture and Peyronie’s disease and can coexist.^9^

Current treatments include conservative and surgical options if conservative therapies are unsuccessful. Nonoperative management includes physical therapy, orthotics, radiotherapy, nonsteroidal antiinflammatory drugs, and intralesional corticosteroid injection. Surgical resection is reserved for tumors that have failed conservative management. Such masses are generally large, painful, or refractory to orthotics; treatment ranges from local excision to plantar fasciectomy.^10^ Surgical complications include high recurrence rates, wound infections, delayed healing, and nerve damage.^10^

Injection therapy with collagenase has been approved by the US Food and Drug Administration (FDA) for Dupuytren’s contracture and Peyronie’s plaques for over 10 years. Collagenases are enzymes that hydrolyze collagen, thereby softening and shrinking masses primarily composed of collagen aggregates.

Although plantar fibromatosis is histologically identical to Dupuytren’s contracture and Peyronie’s disease, collagenase injection treatment for plantar fibromatosis to date has not been FDA-approved because of the lack of adequate clinical trials to demonstrate efficacy and safety. A few published case reports of off-label use of collagenase injection therapy support the effectiveness of this treatment.^3,8^

## Case report

A 42-year-old man presented to the foot and ankle surgery practice with a painful, palpable, oval-shaped, golf ball–sized mass on the medial side of the plantar arch of his left foot.

**Figure 1.**
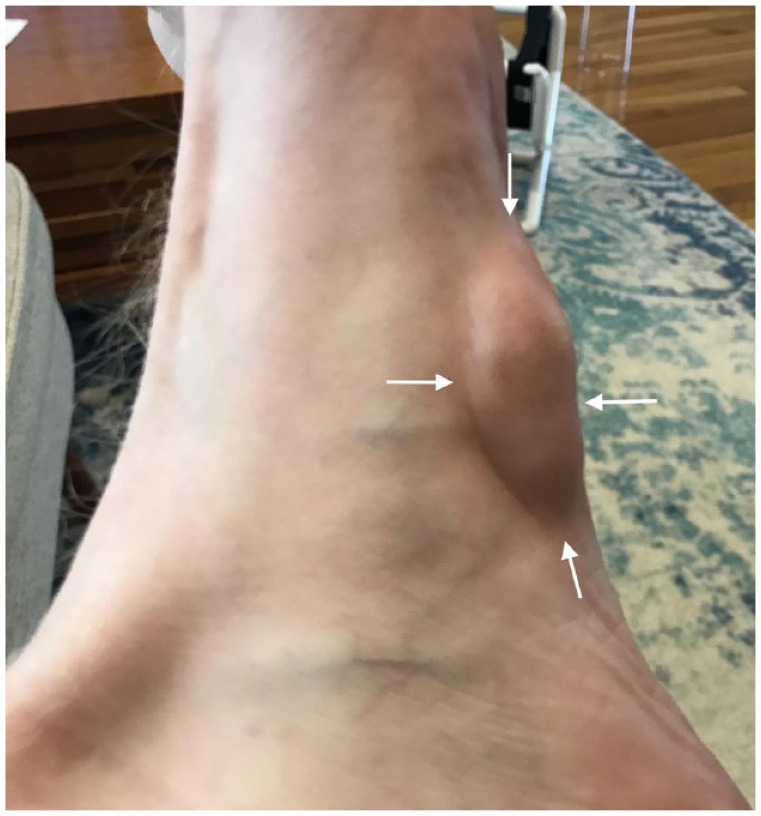
Clinical photograph of the patient’s left foot at initial presentation to our institution, demonstrating a visibly protruding plantar mass (white arrows), recurrent after surgical resections and prior to therapeutic collagenase (Xiaflex) injection.

Physical examination demonstrated a visibly protruding, firm, well-defined mass in the subcutaneous soft tissues of the left plantar arch deep to a well-healed scar from prior surgical resection of a plantar fibroma (Figure 1). Three additional small asymptomatic plantar masses were palpated on the right foot. The patient had no findings of coexisting Dupuytren’s contracture or Peyronie’s disease. The patient has no other medical conditions associated with risk for plantar fibromatosis such as obesity, diabetes, alcohol or tobacco use, or peripheral vascular disease.

The plantar masses had developed after prior surgical resection of a large symptomatic mass on his left foot 13 years prior to presentation and a revision resection surgery 9 years prior to presentation. The mass reappeared within 6 months of the second procedure, which had been complicated by severe methicillin-resistant *Staphylococcus aureus* infection. The prior clinical records were unavailable.

**Figure 2.**
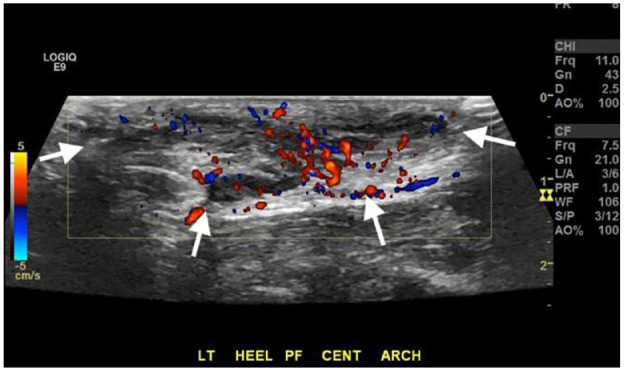
Static longitudinal sonographic image of the palpable 4.1-cm plantar foot mass (white arrows) located within the central band of the plantar fascia with associated hyperemia as seen with color Doppler, imaged prior to therapeutic collagenase (Xiaflex) injection.

The patient was seeking a nonsurgical alternative given his surgical complications. In preparation, diagnostic ultrasonography of the left foot was performed using a GE LOGIQ E9 ultrasound machine with musculoskeletal optimization package and a high-resolution linear transducer (GE L8-18i-D). The area of palpable concern at the plantar fascia demonstrated an ill-defined elongated solid hypoechoic mass arising from the central band. The mass measured 4.1 cm at maximal length, 2.2 cm in width, and 1.0 cm in thickness. The mass was infiltrative into the adjacent subcutaneous soft tissues and skin in the area of the superficial surgical scar. Moderate hypervascularity of the mass was seen with color Doppler ultrasonography (Figure 2).

Ultrasound-guided percutaneous injection therapy with collagenase Clostridium histolyticum was deemed feasible. The patient was counseled regarding the off-label usage of the injectable medication. He consented to the procedure.

One vial containing freeze-dried 0.9 mg collagenase (Xiaflex; Endo Pharmaceuticals Inc) was reconstituted in a proprietary diluent according to manufacturer’s instructions.^11^ The total dose of 0.9 mg of collagenase was divided into 2 equal doses of 0.45 mg each.

**Figure 3.**
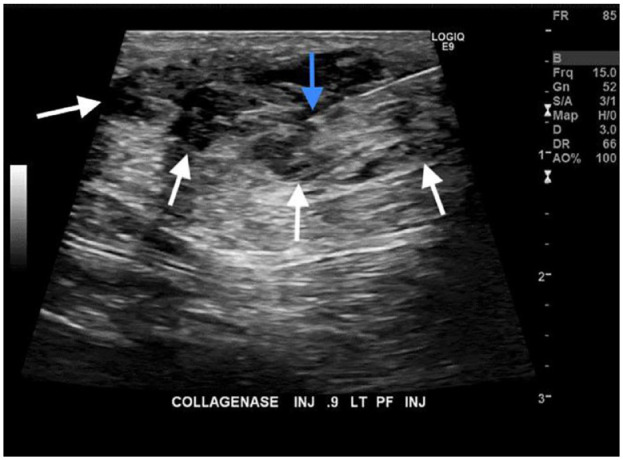
Ultrasonographic image during ultrasonographically-guided fine needle placement (needle tip: blue arrow) into the center of the mass (mass outlined by white arrows) and injection of collagenase (Xiaflex) into the plantar fibroma.

Using sterile technique and local anesthetic with buffered 1% lidocaine, a 25-gauge hypodermic needle was advanced through the skin into the fibroma from a plantar approach under ultrasonographic guidance. The needle tip was first directed into the proximal aspect of the fibroma and 0.45 mg was infiltrated into the midportion of the lesion. The needle was then repositioned into the distal aspect of the fibroma and the remaining 0.45 mg was injected, again at middepth of the mass and ensuring a safety margin of 3 to 4 mm to avoid leakage of collagenase into surrounding soft tissues. Visualization of the needle and the progress of injections were ultrasonographically monitored throughout the procedure to ensure containing the medication within the lesion (Figure 3). After completion of the injection, the needle was withdrawn and a bandage applied.

The patient tolerated the procedure well and there were no immediate complications.

Postprocedural instructions included nonweightbearing and resting of the left foot with intermittent application of external cooling packs for the remainder of the day. After 24 hours, the patient was able to ambulate with the left foot immobilized in a walking boot for 4 weeks. Nonprescription oral analgesics such as acetaminophen for postprocedural pain control were permissible as needed.

Four weeks after the procedure, the patient presented for clinical follow-up. The patient reported significant reduction of symptoms with greater than 50% reduction in pain as compared to pretreatment. The mass had reduced to less than 50% of pretreatment size, and the patient had improved comfort in shoe wear. The patient reported noticeable size improvement 9 days after the procedure with continued gradual decrease of size of the plantar mass and reduction of foot pain over 3 months. The walking boot was discontinued and the patient returned to normal physical activities as tolerated.

A follow-up ultrasonography was performed 3 months after the first injection. A residual but less bulky lesion of plantar fibromatosis at the medial aspect of the central band was demonstrated, measuring 2.5 cm in length, 1.1 cm in width, and 0.4 cm in thickness, indicating partial response to prior injection therapy. Repeat injection therapy with collagenase into the fibroma was performed using similar technique as the first injection treatment, this time with a 0.6-mg dose. The patient followed an identical postprocedure protocol as the first injection. There were no complications.

**Figure 4.**
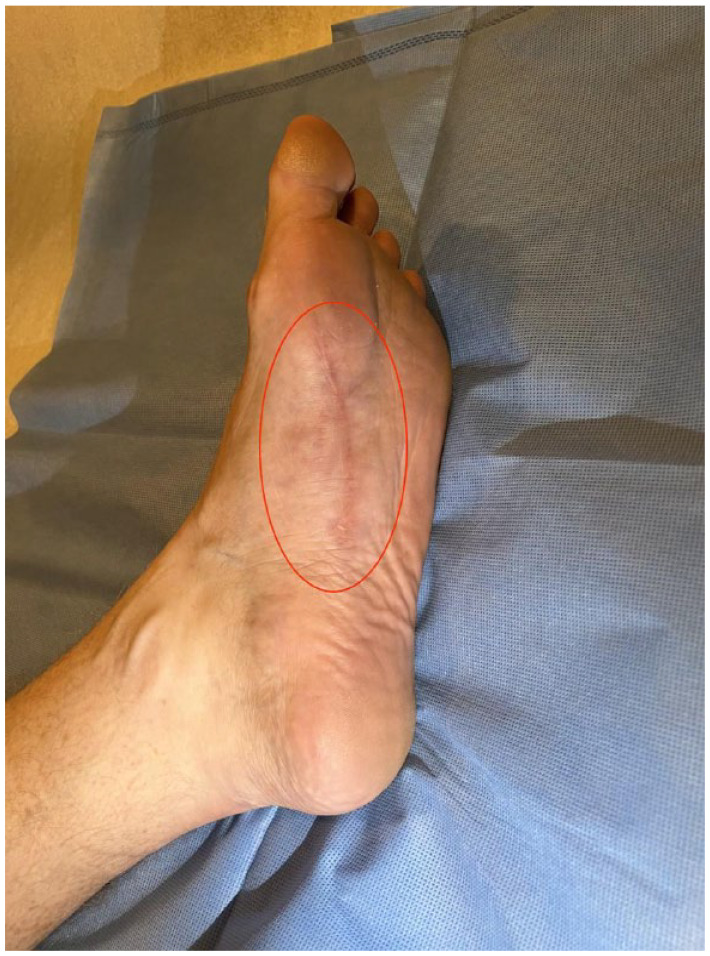
Six-month follow-up clinical photograph post 2 injections with collagenase demonstrating near normal contour of the plantar arch with resolution of the prior plantar mass (red oval outline) and well-healed superficial postsurgical scar.

The patient presented 4 weeks after the second injection therapy for clinical follow-up. At the time, the patient reported near complete resolution of local pain and significant reduction of the palpable mass with clinically essentially normal contour of the plantar surface of the left foot (Figure 4).

**Figure 5.**
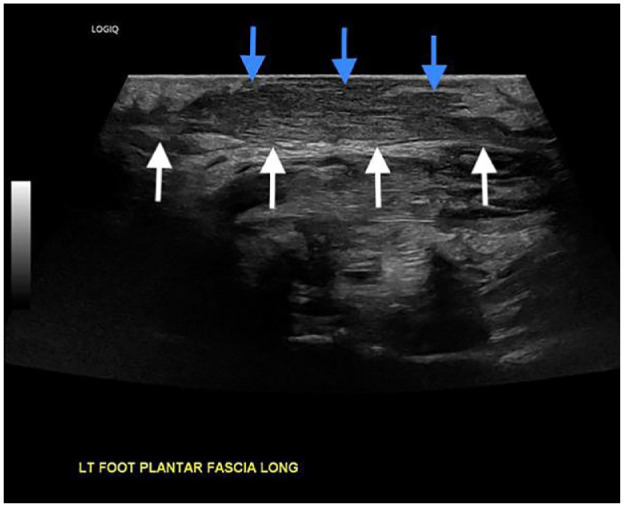
Six-month follow-up static longitudinal sonographic image of the plantar fascia (white arrows) at the location of the prior mass, demonstrating near complete resolution of the plantar mass with residual superficial postsurgical scarring (blue arrows) present.

**Figure 6.**
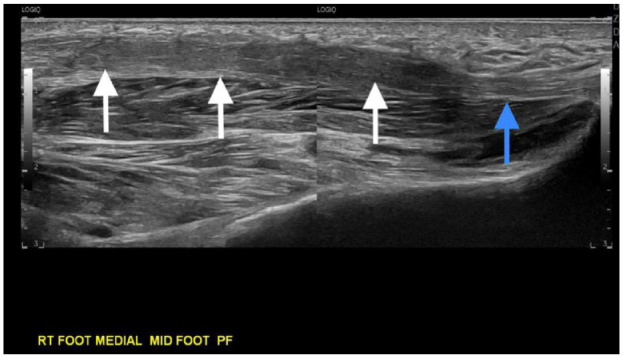
Twelve-month follow-up static composite longitudinal sonographic image of the plantar fascia at the location of the prior mass, demonstrating complete resolution of the plantar mass with mild residual overall thickening of the plantar fascia (white arrows) present. For comparison, the normal, previously uninvolved proximal plantar fascia is seen to the right of the image (blue arrow).

**Figure 7.**
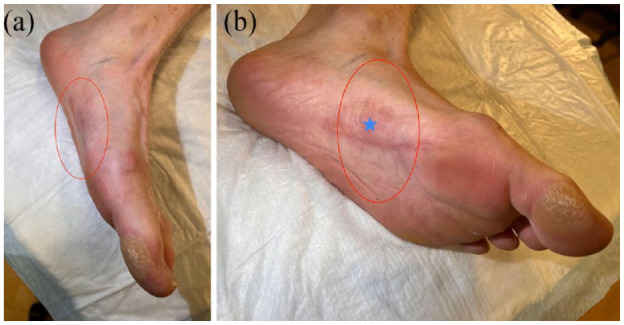
(A, B) Twelve-month follow-up clinical photographs in 2 projections following collagenase injection therapy demonstrate a sustained therapy response with essentially normal contour of the plantar arch of the foot and no recurrence of the plantar mass (red oval outline). Skin discoloration (blue star) is related to remote prior postsurgical and postinfectious scarring of skin and subcutaneous soft tissues.

Ultrasound follow-up at 6 months (Figure 5) and 12 months (Figure 6) from first injection with collagenase showed that the original plantar fibroma clinically (Figure 7) and ultrasonographically (Figure 6) had resolved without a measurable residual abnormality.

The patient was satisfied with the clinical outcome and called it a “resounding success.” He has been able to resume all of his daily activities without limitations.

## Discussion

This case report documents complete resolution of a plantar fibroma through ultrasound-guided collagenase Clostridium histolyticum injection therapy in a patient with recurrent plantar fibromatosis after 2 surgical resections, one of which was complicated by wound infection. Reports documenting the effectiveness, safety, and clinical outcomes of collagenase Clostridium histolyticum injection therapy in patients with symptomatic plantar fibromatosis have been limited to case reports in both adults and children, with varying success.^3,8^ This report is the first to demonstrate the utility of ultrasound guidance for accurate and safe delivery of collagenase injection therapy.

There is known histologic similarity of plantar fibromatosis, Dupuytren’s contracture, and Peyronie’s disease. We hypothesize that therapy with collagenase Clostridium histolyticum injection, proven to be effective in the latter two conditions, could be effective in the treatment of plantar fibromatosis.

Hurst et al^5^ studied 308 patients with diagnosis of Dupuytren’s contracture who were randomized into 204 patients receiving collagenase Clostridium histolyticum injection therapy and 108 receiving placebo injections. The study demonstrated that collagenase injection therapy significantly reduced contractures of the fingers and improved the range of motion in joints affected by advanced Dupuytren’s disease. Similar results were reported by Badalamente et al in a smaller population of 35 patients.^1^ A review conducted by Brazzelli et al provided evidence that collagenase injection therapy was significantly better than placebo for the treatment of Dupuytren’s contracture.^2^ Lauritzson et al demonstrated that clinical improvement after treatment with collagenase Clostridium histolyticum was maintained in 72% of the treated hands up to 2 years post collagenase injection.^6^

The effectiveness of collagenase injections for plantar fibromatosis has been reported in 2 case reports involving adult patients. De Vitis et al^1^ reported a 59-year-old male patient with multiple risk factors and bilateral disease, for whom collagenase injection was performed directed into the mass by clinical palpation using a dose of 0.58 mg, achieving a recurrence-free response for at least 1 year on reported follow-up. A second case report describes a 20-year-old female with recurrent left plantar fibromatosis following conservative and surgical treatments. Collagenase was injected under palpation of the mass at a dose of 0.58 mg each and achieved a symptom-free response up to 84 months postinjection.^8^

Our patient was treated with a higher dose of collagenase (0.9 mg) because of the large size of the mass. Reported adverse reactions of collagenase injections include regional tendon or ligament rupture, local wound healing complications such as skin laceration, hematoma and swelling, and complex regional pain syndrome.^8^ The injection technique used for this patient included ultrasound guidance and targeted the injection into the center of the mass, ensuring a safety margin of 3 to 4 mm to the periphery of the mass to avoid local complications.

Percutaneous needle fasciotomy in Dupuytren’s contracture has also been suggested to be as effective as collagenase injections, but at significantly lower cost. This has not been studied in plantar fibromatosis. However, our patient had a large mass, and the effectiveness of percutaneous needle fasciotomy was doubtful.

## Summary/Conclusion

We demonstrate successful ultrasound-guided injection therapy of collagenase Clostridium histolyticum in an outpatient setting for a patient with two prior surgeries and recurrent, symptomatic plantar fibromatosis. Clinical trials with larger patient cohorts are necessary to further study the utility of this treatment for plantar fibromatosis, as it may be a viable, nonsurgical treatment option for this difficult-to-treat condition.

## Supplemental Material

sj-pdf-1-fao-10.1177_24730114231201161 – Supplemental material for Ultrasound-Guided Collagenase Injection Therapy of Recurrent Plantar Fibromatosis: A Case Report Click here for additional data file. Supplemental material, sj-pdf-1-fao-10.1177_24730114231201161 for Ultrasound-Guided Collagenase Injection Therapy of Recurrent Plantar Fibromatosis: A Case Report by Antje L. Greenfield, Adan Bello Baez and Casey Jo Humbyrd in Foot & Ankle Orthopaedics
